# Phylogenetic Position of *Shiraia*-Like Endophytes on Bamboos and the Diverse Biosynthesis of Hypocrellin and Hypocrellin Derivatives

**DOI:** 10.3390/jof7070563

**Published:** 2021-07-14

**Authors:** Xin Tong, Qiu-Tong Wang, Xiao-Ye Shen, Cheng-Lin Hou, Paul F. Cannon

**Affiliations:** 1 College of Life Science, Capital Normal University, Xisanhuanbeilu 105, Haidian, Beijing 100048, China; tongxin1112@126.com (X.T.); 2190801014@cnu.edu.cn (Q.-T.W.); 2 Royal Botanic Gardens, Kew, Surrey TW9 3AB, UK; p.cannon@kew.org

**Keywords:** shiraiaceae, endophyte, morphology, secondary metabolites, new taxa

## Abstract

The main active ingredients of the fruiting bodies of *Shiraia bambusicola* and *Rubroshiraia bambusae* are Hypocrellins, belonging perylenequinones with potential photodynamic activity against cancer and microbial diseases. However, the strains of *S. bambusicola* and *R. bambusae* do not produce hypocrellins in culture, so resource exploitation of natural products was seriously restricted. In this study, a series of novel *Shiraia*-like fungal endophyte strains, with varying sporulation ability and synthesizing diverse secondary metabolites, was isolated from different bamboos. Based on phylogenetic analyses and morphological characteristics of the endophytes, *Pseudoshiraia conidialis* gen. et sp. nov. is proposed. The secondary metabolites of different fruiting bodies and strains have been comprehensively analyzed for the first time, finding that the endophytic strains are shown not only to produce hypocrellins, but also other perylenequinonoid compounds. It was noteworthy that the highest yield of total perylenequinone production and hypocrellin A appeared in *P. conidialis* CNUCC 1353PR (1410.13 mg/L), which was significantly higher than any other wild type *P. conidialis* strains in published reports. In view of these results, the identification of *Shiraia*-like endophytes not only confirm the phylogenetic status of similar strains, but will further assist in developing the production of valuable natural products.

## 1. Introduction

Hypocrellins belong to the perylenequinonoid family of compounds. They are very important photosensitizers and have attracted broad attention because of their light-induced antitumour, antifungal and antiviral activities [1,2,3,4,5,6,7]. In China, hypocrellins have been used medicinally to treat skin diseases for many years [8]. In recent years, hypocrellins and their derivatives have been incorporated into polymer micelles or nanoparticles for the treatment of methicillin-resistant *Staphylococcus aureus* infections [9] and cancer therapy [10,11,12]. In addition to benefiting the pharmaceutical industry, hypocrellins also have extensive potential applications in the agricultural, cosmetic, food and feed industries [13,14,15].

**Figure 1:**
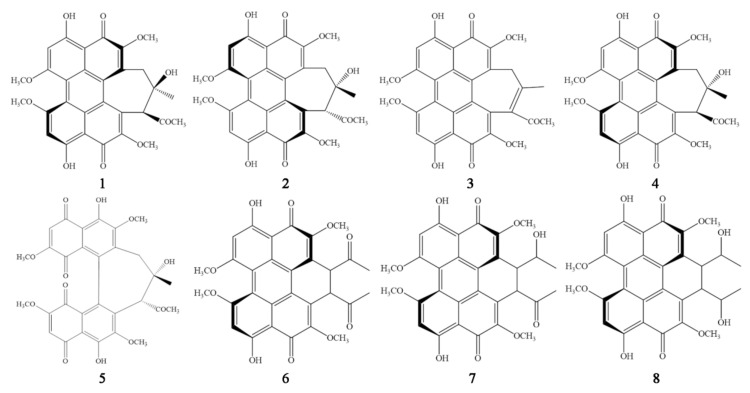
Chemical structures of hypocrellin (**1**), hypocrellin A (**2**), hypocrellin B (**3**), shiraiachrome A (**4**), hypocrellin D (**5**), elsinochrome A (**6**), elsinochrome B (**7**) and elsinochrome C (**8**).

The hypocrellin cluster consists of five main compounds—hypocrellin (1), hypocrellin A (2), hypocrellin B (3), shiraiachrome A (4), and hypocrellin D (5), and their structures are shown in Figure 1. There is some confusion in the literature over the nomenclature of these compounds, and in this paper the names as defined by Al Subeh et al. are followed [16]. Hypocrellin (1), hypocrellin A (2) and hypocrellin B (3) were first isolated from traditional Chinese medicinal products, which were called “Zhuhongjun” or “Zhuxiaorouzuojun” [1,17,18,19], and were identified as *Hypocrella bambusae* (Berk. & Broome) Sacc. by Liu [20]. Subsequent studies reported that extracts of ascostromata of *Shiraia bambusicola* Henn. also contained hypocrellin A (2) and hypocrellin B (3), as well as shiraiachrome A (4) and hypocrellin D (5) [21,22,23].

However, the taxonomic status of hypocrellin producing species remained confused. Liu et al. introduced the family Shiraiaceae (Pleosporales) Y.X. Liu, Zi Y. Liu & K.D. Hyde to accommodate genus *Shiraia* Henn [24]. More recently, Dai et al. described a new genus *Rubroshiraia* D.Q. Dai & K.D. Hyde in Shiraiaceae based on morphological characteristics and phylogenetic analysis, and concluded that the traditional Chinese medicine which was called “Zhuhongjun” or “Zhuxiaorouzuojun” in Chinese should be assigned to the new species *R. bambusae* D.Q. Dai & K.D. Hyde rather than the unrelated *Hypocrella bambusae* (Berk. & Broome) Sacc [25].

*Shiraia*-like endophytes associated with plants have been identified and shown to produce hypocrellins [16,26,27,28,29]. Based on phylogenetic analysis, these strains generally clustered in the family Shiraiaceae [25,26], but their explicit taxonomic status could not be established due to the lack of morphological characteristics.

Since 2008, we have been characterizing *Shiraia*-like endophytes from bamboos, and have explored several interesting strains with conidial production, and diverse natural products. The aim of this study is to accurately establish the taxonomic status of *Shiraia*-like endophytes based on morphological characteristics and phylogenetic analysis, and comprehensively analyze the secondary metabolites of these strains.

## 2. Materials and Methods

### 2.1. Isolates

Fruit bodies of *Shiraia bambusicola* were collected from Hangzhou, Zhejiang, China and those of *Rubroshiraia bambusae* from Yulong County, Yunnan, China. Fungal endophytes were isolated from asymptomatic tissues of bamboos (Poaceae: Bambusoideae) in various localities in China (Table 1). The methods of isolation are described in Shen et al. [30] and Zhou et al. [31]. All isolates were cultured on Potato Dextrose Agar (PDA, containing 200 g/L potato, 20 g/L dextrose and 20 g/L agar) at 25 °C for 14 days to observe the morphology. The specimens were deposited in the Fungarium of the College of Life Science, Capital Normal University, Beijing, China (BJTC) and the China Forest Biodiversity Museum of the Chinese Academy of Forestry (CAF), and ex-type living cultures were deposited in the China Forestry Culture Collection Center (CFCC) and Capital Normal University Culture Collection Center (CNUCC).

### 2.2. Morphological Analysis

Measurements and photographs of characteristic structures were made according to methods described by Liu et al. [32], and for each structure 30 measurements were made. Microscopic preparations were made in clear H_2_O, observed and photographed using a Nikon SMZ-1000 dissecting microscope (DM), an OLYMPUS light microscope (LM) or a Hitachi S-4800 scanning electron microscope (SEM). Colony characters and pigment production on PDA incubated at room temperature were noted after 14 d. Colony colors were taken from ColorHexa (https://www.colorhexa.com/, accessed on 18 December 2020). Growth rates were measured after 7 and 14 d.

### 2.3. DNA Extraction, PCR Amplification and Sequencing

Genomic DNA extraction was conducted according to Shen et al. [30]. Five loci including the 5.8S nuclear ribosomal gene with the two flanking internal transcribed spacers (ITS), the large subunit rDNA (LSU), the small subunit rDNA (SSU), the translation elongation factor 1-α gene region (TEF1) and the RNA polymerase II second largest subunit (RPB2) were amplified and sequenced using the primer pairs ITSIF [33] + ITS4 [34], LR0R + LR5 [35], NS1 + NS4 [34], EF1-983F + EF1-2218R [36] and fRPB2-5f and fRPB2-7cr primers [37], respectively. The PCR mixture (25 µL, total volume) contained 2 µL template, 1 µL of each primer (10 mM each), 12.5 µL 2 × M5 HiPer Taq PCR mix (Mei5Bio, Beijing, China) and 8.5 µL ddH_2_O. The PCR amplification protocols are given in Table 2. The purified PCR products were sequenced by Zhongkexilin Biotechnology Co., Ltd. (Beijing, China).

### 2.4. Phylogenetic Analysis

The new sequences were submitted to the GenBank database and other sequences included in this study were downloaded from GenBank (Table 1) based on recent publications [25,38]. The DNA sequences generated with forward and reverse primers were aligned to obtain consensus sequences using EditSeq version 5.00. A partition homogeneity test was done to determine the congruence of gene fragments [39,40]. Subsequent alignments were generated using online MAFFT tools (https://www.ebi.ac.uk/Tools/msa/mafft/, accessed on 24 November 2020), and edited using Gblocks 0.91b (http://www.phylogeny.fr/one_task.cgi?task_type=gblocks, accessed on 24 November 2020), selecting all options for a less stringent selection.

Maximum parsimony (MP) analysis was performed on the multi-locus alignment including two loci (ITS and LSU) with PAUP v.4.0b10 [41], using the heuristic search option with tree bisection and reconstruction (TBR) branch swapping and 1000 random sequence additions. Maxtrees were 1000, branches of zero length were collapsed and all multiple parsimonious trees were saved. Clade stability was assessed in a bootstrap analysis with 1000 replicates, each with 10 replicates of random stepwise addition of taxa. Tree statistics (TL), consistency index (CI), retention index (RI), rescaled consistency index (RC) and homoplasy index (HI) as the descriptive tree statistics were calculated for the generated trees.

For the Bayesian analysis, a Markov Chain Monte Carlo (MCMC) algorithm was conducted to reconstruct the single locus and multi-locus phylogenetic trees with Bayesian posterior probabilities in MrBayes v. 3.1.1 [42]. For the Bayesian analysis, models of nucleotide substitution were determined by MrModeltest v.2.3 [43] for each gene and included in the analyses (ITS, LSU, TEF and TUB2: GTR + I + G, SSU: HKY + I). The analyses of four chains were conducted for 10,000,000 generations with the default settings and sampled every 100 generations, halting the analyses at the average standard deviation of split frequencies of 0.01. The first 25% trees were discarded as the burn-in phase of the analyses and the posterior probabilities (PP) were obtained from the remaining trees.

Maximum likelihood (ML) analysis of the dataset was carried out using RAxML 8.0.14 [44,45,46] and the GTRGAMMI substitution model with parameters unlinked. The ML bootstrap replicates (1000) were computed in RAxML using a rapid bootstrap analysis and search for the best-scoring ML tree.

**Figure 2:**
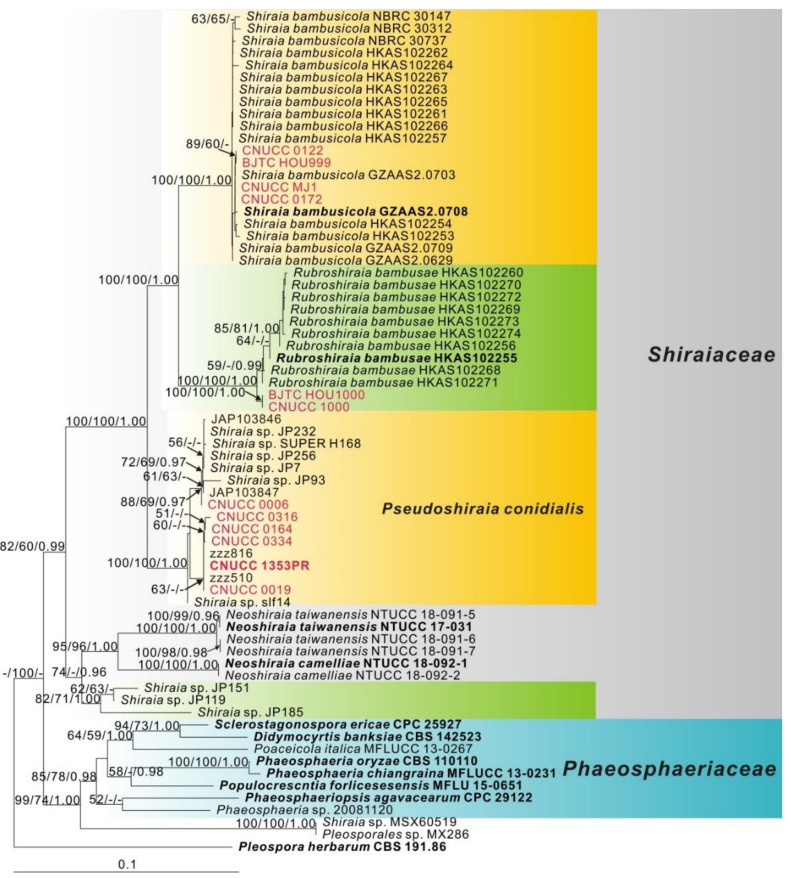
The phylogenetic tree based on the concatenated alignment of four molecular markers (ITS, LSU, SSU, TEF and RPB2) evaluated using RAxML. The new isolates are shown in red. Numbers of ex-holotype or ex-epitype strains are emphasized in bold. ML bootstrap values (MLBS) ≥ 50%, MP bootstrap values (MPBS) ≥ 50% and Bayesian posterior probabilities (PP) ≥ 0.95 are at each node. The scale bar indicates the number of estimated substitutions per site. *Pleospora herbarum* (CBS 191.86) was used as outgroup for rooting the tree.

Trees were viewed in Treeview [47] and edited in Coreldraw v.X4 (Corel Corporation, Canada). ML bootstrap values (MLBS) and MP bootstrap values (MPBP) equal to or greater than 50% and Bayesian posterior probability (PP) equal to or greater than 0.95 are given at each node (Figure 2). The combined alignment and phylogenetic tree were submitted at TreeBASE (www.treebase.org, accessed on 24 November 2020; study S28492).

### 2.5. Submerged Cultivation and Secondary Metabolite Extraction

The endophytic fungi isolates were cultured on PDA at 26 °C for 7 days. Based on colony colour, three strains representing different morphs were selected for further experiments. Five plugs (5 mm in diameter) of growing culture plus the adhering mycelium were added to 250 mL Erlenmeyer flasks containing 150 mL of Potato Dextrose Broth media (PDB, containing 200 g/L potato and 20 g/L dextrose). All liquid cultures were kept at 26 °C for 10 d with shaking (180 rpm).

Fresh mycelia of the fungal strains *Shiraia bambusicola* CNUCC 0172, CNUCC 0122 and CNUCC MJ1 were cultured on PDA at 26 °C for 10 days. Five plugs (5 mm in diameter) of growing culture plus the adhering mycelium were subsequently added to 150 mL PDB. The liquid cultures were kept at 26 °C for 10 d with shaking (180 rpm).

Fresh mycelia of the fungal strain *Rubroshiraia bambusae* CNUCC 1000 were cultured on PDA at 16 °C for 30 days. Five plugs (5 mm in diameter) of growing culture plus the adhering mycelium were subsequently added to 150 mL PDB. The liquid cultures were kept at 16 °C for 40 d with shaking (180 rpm).

The fermented mycelia of each fungus were filtered and dried at 45 °C. The dry powder (0.1 g) of ascostromata and mycelia of *S. bambusicola* and *R. bambusae* was accurately weighed and ultrasonic extracted for 30 min with 5 mL methanol. The dry powder of endophytic mycelia was treated in the same manner.

### 2.6. HPLC-DAD-MS Analysis

HPLC-DAD-MS analysis was performed using a Shimadzu LC-20AD liquid chromatography (LC) system coupled with a diode array detector (DAD) and an electrospray ionization-ion-trap-time-of-flight (ESI-IT-TOF) mass spectrometer (MS) (Shimadzu, Kyoto, Japan). For analytical purposes, a Kromasil 100-5 C18 (250 × 4.6 mm, 5 µm) column was used. The mobile phase was composed of water containing 0.1% formic acid (A) and methanol (B), and the gradient of eluent B started at 5% and gradually increased to 90% over 90 min at a flow rate of 1 mL/min. The MS conditions refer to Niu et al. [48].

Standards of hypocrellin A (HA), hypocrellin B (HB) and shiraiachrome A (SA), purity ≥ 98% (HPLC), were purchased from Biopurify Phytochemicals Ltd. Standards of elsinochrome A (EA), elsinochrome B (EB) and elsinochrome C (EC), purity ≥ 98% (HPLC), were purchased from Hangzhou Viablife Biotech CO., Ltd. An Agilent 1200 HPLC-DAD system was used to analyze the perylenequinonoid compounds, which was equipped with a Kromasil 100-5 C18 (250 × 4.6 mm, 5 µm) column. The column was maintained at 35 °C. The mobile phase was composed of water containing 0.1% phosphoric acid (A) and methanol (B) at a flow rate of 1 mL/min with the following gradient: 0–10 min, 60% B; 10–15 min, 60–70% B; 15–25 min, 70% B; 25–45 min, 70–75% B; 45–60 min, 75–100% B; 60–80 min, 100% B. The UV-Vis spectrum was recorded at 200–800 nm. All sample solutions were filtered by a membrane (0.22 μm) prior to analysis. For each sample, the injection volume was 10 µL, and the external standard method [29] was applied for the quantitative analysis, using a detection wavelength of 460 nm.

## 3. Results

### 3.1. Phylogeny

The multi-locus phylogenetic analysis included 68 ingroup samples, and used *Pleospora herbarum* (CBS 191.86) as outgroup. The dataset of five loci comprised 3 831 characters including the alignment gaps, of which 622 characters were parsimony-informative, 172 parsimony-uninformative and 3037 constant. A best scoring RAxML tree is shown in Figure 2, the maximum parsimony and Bayesian tree confirmed the tree topology obtained with maximum likelihood.

The results showed that among 14 strains isolated in this study, 10 strains were clustered together with endophyte group A as listed in Dai et al. [25], forming a highly supported clade (BS = 100, BP = 100, PP = 1.00). The strains CNUCC 0122, CNUCC 0172 and CNUCC MJ1 together with BJTC HOU999 were clustered in the clade of *S. bambusicola*. The strains CNUCC 1000 together with BJTC HOU1000 were clustered in the clade of *R. bambusae*.

### 3.2. Taxonomy

Based on phylogenetic analyses and morphological characteristics, a novel species belonging to a new genus was recognized in this study.

Shiraiaceae Y.X. Liu, Zi Y. Liu & K.D. Hyde, Phytotaxa 103(1): 53 (2013).

**Figure 3:**
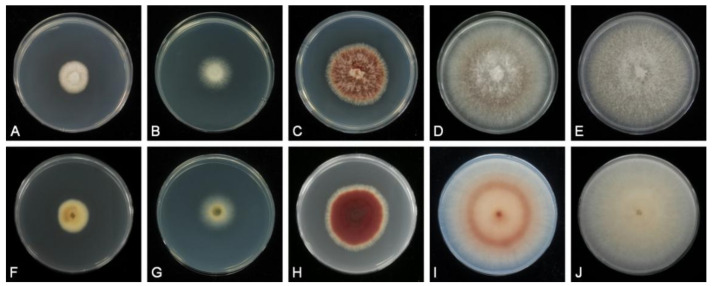
Colony morphology of cultures from *Shiraia*-like fungi. (**A**,**F**). Culture derived from a fruiting body of *Shiraia bambusicola* BJTC HOU999. (**B**,**G**). Culture derived from a fruiting body of *Rubroshiraia bambusae* BJTC HOU1000. (**C**,**H**). *Pseudoshiraia conidialis* CNUCC 1353PR. (**D**,**I**). *Pseudoshiraia conidialis* zzz816. (**E**,**J**) *Pseudoshiraia conidialis* JAP103846.

**Figure 4:**
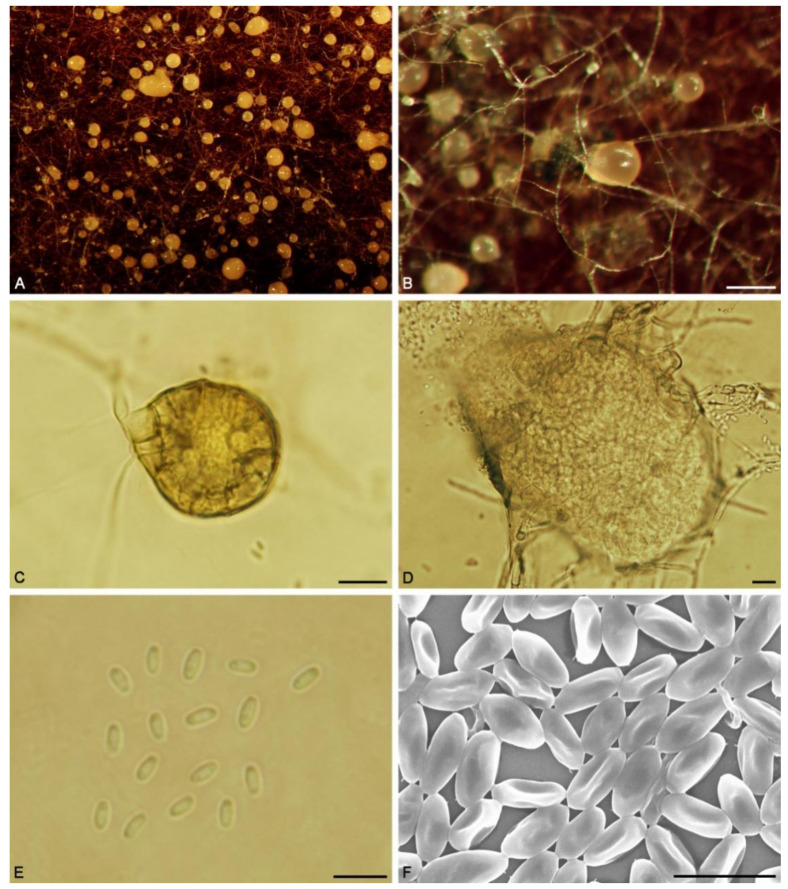
*Pseudoshiraia conidialis* (CNUCC 1353PR) (**A**). A mass of conidiomata. (**B**). Conidiomata on PDA. (**C**,**D**). Conidiomata. (**E**). Conidia. (**F**). SEM of conidia. Bars: B = 200 μm; C–D = 10 μm; E–F = 5 μm.

*Pseudoshiraia conidialis* C.L. Hou, Q.T. Wang & P.F. Cannon gen. et sp. nov. (Figure 3 and Figure 4).

MycoBank MB840497 (genus)

MycoBank MB840566 (species)

Etymology. The name reveals that this species can produce hypocrellins like the species in genus *Shiraia*, but this species can only be found in an anamorphic stage at present.

Typification. CHINA, Yunnan province, on tissues of bamboos, May 2018, Q.T. Wang & C.L. Hou (holotype CAF80003). Culture ex-type CNUCC 1353PR = CFCC 55715.

Diagnosis. *Pseudoshiraia conidialis* differs from *Shiraia bambusicola* by small, cylindrical or ellipsoidal conidia, without septa.

Description. Sexual morph unknown. On PDA: conidiomata erumpent through mycelium surface, brown, rough and hard, multi-loculate, 150−500 × 175−625 μm ($\bar{x}$ = 375 × 450 μm, *n* = 30). Conidiomatal wall of thick-walled angular cells, 5−8 μm in diam. Conidiogenous cells 4−5 × 2.5−3 μm, enteroblastic, phialidic, hyaline, ampulliform, discrete, smooth. Conidia 1.5−2 × 2−3 μm ($\bar{x}$ = 1.6 × 2.4 μm, *n* = 30), hyaline, aseptate, cylindrical to ellipsoidal, smooth-and thin-walled.

Culture characteristics. Colonies on PDA 12–15 mm diam in 7 d (56–59 mm in 14 d), with entire margin, surface very dark red (670a00), entirely covered with sparse white aerial mycelium and masses of pure (or mostly pure) orange (e6ac00) conidiomata. Reverse dark red (8a0022).

Note. The endophytic strains isolated from bamboos in this study and *Shiraia*-like endophytic strains which produce hypocrellins clustered together with high support values. This clade showed a close relationship with *Shiraia bambusicola* and *Rubroshiraia bambusae*. *S. bambusicola* is known to produce both sexual and asexual morphs, while *R. bambusae* only produces a sexual morph [25]. The conidia of *P. conidialis* and *S. bambusicola* are quite different. Those of *P. conidialis* are cylindrical or ellipsoidal, without septa, 1.5−2 × 2−3 μm ($\bar{x}$ = 1.6 × 2.4 μm, *n* = 30); while those of *S. bambusicola* are fusiform and muriform with 15–18 transverse septa, measuring 60−80 × 19−25 μm ($\bar{x}$ = 75.4 × 23.1 μm, *n*=20) [25]. Therefore, based on morphological characteristics and phylogenetic analysis, a new genus *Pseudoshiraia* may be established to accommodate the new species *P. conidialis*.

Other material examined. Guangxi Zhuang Autonomous Region, on seeds of *Phyllostachys edulis* (Carriere) J. Houzeau, Dec. 2006, C.L. Hou, culture zzz816.

### 3.3. Hypocrellin Identification and Production

**Figure 5:**
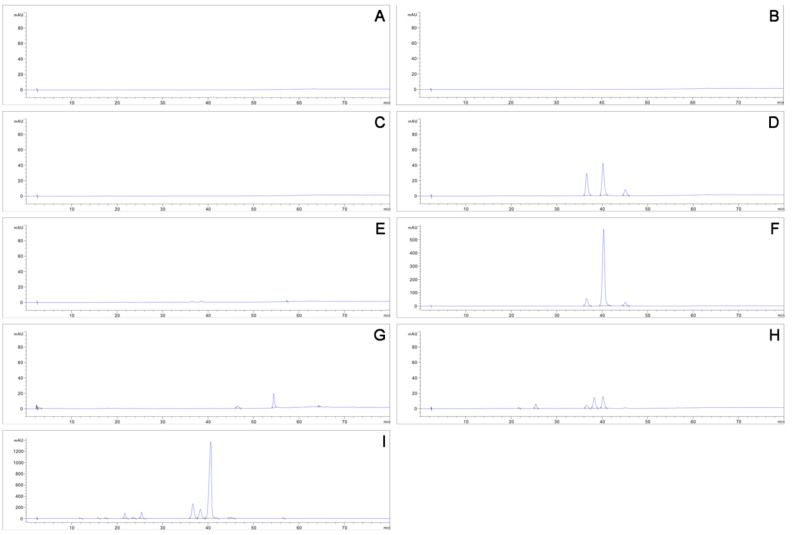
HPLC chromatograms of the samples. (**A**) mycelia of *S. bambusicola* CNUCC MJ1. (**B**) mycelia of *S. bambusicola* CNUCC 0122. (**C**) mycelia of *S. bambusicola* CNUCC 0172. (**D**) fruit body of *S. bambusicola* BJTC HOU999. (**E**) mycelia of *R. bambusae* CNUCC 1000. (**F**) fruit body of *R. bambusae* BJTC HOU1000. (**G**) mycelia of *P. conidialis* JAP103846. (**H**) mycelia of *P. conidialis* zzz816. (**I**) mycelia of *P. conidialis* CNUCC 1353PR.

Six perylenequinonoid compounds were identified and quantified compared with UV-Vis spectra, MS spectra and retention time (R_t_) of standards (Supplementary Table S1). The HPLC chromatograms of extracts derived from fruit bodies of *S. bambusicola* BJTC HOU999 and *R. bambusae* BJTC HOU1000, and mycelia of *S. bambusicola* CNUCC MJ1, CNUCC 0122, CNUCC 0172, *R. bambusae* CNUCC 1000, *P. conidialis* CNUCC 1353PR, zzz816 and JAP103846 are all shown in Figure 5. The peaks at 40.3 min, 45.1 min, 36.6 min, 38.3 min, 25.4 min and 21.7 min are indicated as HA, HB, SA, EA, EB and EC separately. For each sample, the content of each perylenequinonoid compound was calculated by the standard curves (Table 3 and Table 4). No perylenequinonoid compounds appeared in extracts from mycelia of *S. bambusicola* CNUCC MJ1, CNUCC 0122, CNUCC 0172, *R. bambusae* CNUCC 1000 and *P. conidialis* JAP103846. Those from fruit bodies of *S. bambusicola* BJTC HOU999 and *R. bambusae* BJTC HOU1000 only produced hypocrellins, and that from *R. bambusae* BJTC HOU1000 (65.89 mg/g) produced a higher quantity of hypocrellins than *S. bambusicola* BJTC HOU999 (10.39 mg/g). It is noteworthy that the extracts from *P. conidialis* CNUCC 1353PR contained a large concentration of natural products, and both the total perylenequinonoid compound content (1410.13 mg/L) and that of the single compound HA (677.11 mg/L) showed a substantially higher quantity than any other known wild type strains in public papers [49,50,51,52].

## 4. Discussion

In our previous study, a large number of strains were isolated from *S. bambusicola* and *R. bambusae* (Data not shown). However, hypocrellins were found only in fruiting bodies of *S. bambusicola* and *R. bambusae*, and cultured strains did not produce these chemicals (Figure 3). More recent studies have demonstrated that a few *Shiraia*-like endophytes isolated from bamboo tissues could produce hypocrellins, and even the strains isolated from the stromata of *S. bambusicola* could also produce hypocrellins [27,30,50,53,54,55]. But the taxonomic statuses of these strains were very confusing. Such as the strains *Shiraia* sp. SUPER H168, *Shiraia* sp. slf14, *Shiraia* sp. S9, *S. bambusicola* UV-62 and *S. bambusicola* ZH-5-1 were isolated from bamboo tissues as endophytes or isolated from the stromata of *S. bambusicola* [27,50,53,56,57]. Although they were regarded as *Shiraia* spp., the strains *Shiraia* sp. SUPER H168, *Shiraia* sp. slf14 and *Shiraia* sp. S9 were clustered with *Shiraia*-like endophytes but not with *Shiraia* in the phylogenetic trees (Figure 2) [25,50]. Therefore, the taxonomic statuses of other strains like these need to be re-identified.

Morakotkarn et al. [26] and Dai et al. [25] isolated 22 *Shiraia*-like endophytic strains from bamboo tissues. Based on phylogenetic analysis, these strains were divided into two groups, one which could produce hypocrellins, and the other not. Among them, the group with hypocrellins was clustered with these known production strains (Figure 2), forming a highly supported clade. These belong to the newly described species *Pseudoshiraia conidialis*.

Unfortunately, cultures from most of the corresponding endophytes only had low production yield, and several attempts were made to increase hypocrellins production, such as addition of Triton X-100 surfactant to the submerged cultures [49], exposing cultures of *Shiraia* sp. to light at various wavelengths [58] or light/dark shift [59], or co-cultivation of *Shiraia* sp. with *Pseudomonas fulva* [50,60]. Our previous work [8] attempted to use gamma rays to mutate *P. conidialis* zzz816, boosting the HA production to increase to 414.9%.

The conidium-producing and hypocrellin-generating strain CNUCC 1353PR was isolated and characterized in this study. To the best of our knowledge, hypocrellin production in *P. conidialis* CNUCC 1353PR is significantly higher than other wild type *P. conidialis* strains in public [49,50,51,52]. Through comparative analysis of the metabolites of ascostromata and mycelia of *S. bambusicola* and *R. bambusae*, we discovered that HA, HB and SA only appeared in the ascostromata, but not in mycelia of cultured strains. In addition, the *P. conidialis* strains contain more diverse perylenequinonoid compounds, and in addition to HA, HB and SA, also produced EA, EB and EC; demonstrated by the four peaks (R_t_ 12 min, 16 min, 17.5 min and 23.6 min) in the HPLC chromatogram of the mycelia of *P. conidialis* CNUCC 1353PR (Figure 5I), and the UV-Vis spectra indicated that they all belonged to perylenequinones (Supplementary Figure S1). These results displayed that *P. conidialis* CNUCC 1353PR could be a potential industrial strain for perylenequinone production.

Other sources of perylenequinone production could be found. For example, strain MSX60519, isolated from dry leaf litter, was also found to produce hypocrellins [16], and Li et al. [61] explored an endolichenic strain, *Phaeosphaeria* sp. 20081120, which could produce HA, HB and other perylenequinones. Meng et al. [62] investigated metabolites produced by an endophytic fungus identified as *Penicillium chrysogenum* isolated from *Fagonia cretica*, and also found hypocrellins. However, these strains did not cluster with Shiraiaceae (Figure 2). Although the species of other family may also produce hypocrellins, further studies and verification are needed.

## 5. Conclusions

In this study, a new genus *Pseudoshiraia* in Shiraceae was established, and a series of species with high output of hypocrellins were exploited for the first time. Furthermore *P. conidialis* CNUCC 1353PR contains multiple types of perylenequinones, not only hypocrellins but also elsinochromes.

## Figures and Tables

**Table 1:** GenBank accession numbers of isolates used for phylogenetic construction.

Species	Specimen Voucher	Location	GenBank Accession Numbers				
			ITS	LSU	SSU	TEF	RPB2
*Didymocyrtis banksiae*	CBS 142523 *	Australia	KY979757	KY979812	—	—	KY979850
*Neoshiraia camelliae*	NTUCC 18-092-1 *	China, Taiwan	MT112286	MT071262	MT071213	MT743267	MT513982
	NTUCC 18-092-2	China, Taiwan	MT112287	MT071263	MT071214	MT743268	MT513981
*Neoshiraia taiwanensis*	NTUCC 17-031 *	China, Taiwan	MT112285	MT071261	MT071212	MT114404	MT743276
	NTUCC 18-091-5	China, Taiwan	MT112280	MT150600	MT071207	MT114405	MT434762
	NTUCC 18-091-6	China, Taiwan	MT112309	MT150602	MT071236	MT425440	MT434766
	NTUCC 18-091-7	China, Taiwan	MT112310	MT150601	MT071237	MT425441	MT434765
*Phaeosphaeria chiangraina*	MFLUCC 13-0231 *	Thailand	KM434270	KM434280	KM434289	KM434298	KM434307
*Phaeosphaeria oryzae*	CBS 110110 *	South Korea	KF251186	KF251689	GQ387530	—	KF252193
*Phaeosphaeria* sp.	20081120	China, Yunnan	HQ324780	—	—	—	—
*Pleospora herbarum*	CBS 191.86 *	India	KC584239	DQ247804	GU238232	DQ471090	DQ247794
*Pleosporales* sp.	MX286	—	JQ905814	—	—	—	—
*Phaeosphaeriopsis agavacearum*	CPC 29122 *	Australia	KY173430	KY173520	—	—	KY173591
*Poaceicola italica*	MFLUCC 13-0267	Italy	KX926421	KX910094	KX950409	MG520924	KX891169
*Populocrescentia forlicesesensis*	MFLU 15-0651 *	—	KT306948	KT306952	KT306955	MG520925	—
*Pseudoshiraia conidialis*	zzz816	China, Guangxi	HQ696072	MZ519516	HQ696106	MZ516164	MZ516177
	zzz510	China, Guangxi	HQ696078	MZ519517	MZ519540	MZ516165	—
	CNUCC 0316	China, Guangxi	MZ519529	MZ519521	MZ519536	—	—
	CNUCC 0334	China, Guangxi	MZ519528	MZ519520	MZ519535	MZ516160	—
	CNUCC 0019	China, Guangxi	MZ519532	MZ519523	MZ519538	MZ516162	—
	CNUCC 0164	China, Guangxi	MZ519530	MZ519522	MZ519537	MZ516161	MZ516175
	CNUCC 1353PR *	China, Yunnan	MZ519527	MZ519534	MZ519533	MZ516168	—
	CNUCC 0006	China, Anhui	MZ519531	MZ519524	MZ519539	MZ516163	MZ516176
	JAP103846	Japan	MZ519526	MZ519519	MZ519542	MZ516167	MZ516179
	JAP103847	Japan	MZ519525	MZ519518	MZ519541	MZ516166	MZ516178
	JP93	Japan	AB255277	AB354975	—	—	—
	JP256	Japan	AB354995	AB354980	—	—	—
	JP232	Japan	AB255303	AB354979	—	—	—
	JP7	Japan	AB255241	AB354974	—	—	—
	SUPER H168	China	EU267793	—	EU267792	—	—
*Rubroshiraia bambusae*	HKAS102255 *	China	MK804678	MK804658	MK804704	MK819218	—
	HKAS102256	China	MK804679	MK804659	MK804705	MK819219	—
	HKAS102260	China	MK804680	MK804660	MK804706	MK819220	—
	HKAS102268	China	MK804681	MK804661	MK804707	MK819221	—
	HKAS102269	China	MK804682	MK804662	MK804708	MK819222	—
	HKAS102270	China	MK804683	MK804663	MK804709	MK819223	—
	HKAS102271	China	MK804684	MK804664	MK804710	MK819224	—
	HKAS102272	China	MK804685	MK804665	MK804711	MK819225	—
	HKAS102273	China	MK804686	MK804666	MK804712	MK819226	—
	HKAS102274	China	MK804687	MK804667	MK804713	MK819227	—
	BJTC HOU1000	China, Yunnan	MZ497359	MZ497374	MZ497385	MZ516169	—
	CNUCC 1000	China, Yunnan	MZ497360	MZ497376	MZ497387	MZ516170	MZ516180
*Sclerostagonospora ericae*	CPC 25927 *	South Africa	KX228268	KX228319	—	—	—
*Shiraia bambusicola*	HKAS102253	China	MK804668	MK804648	MK804694	MK819208	MK819228
	HKAS102254	China	MK804669	MK804649	MK804695	MK819209	MK819229
	HKAS102257	China	MK804670	MK804650	MK804696	MK819210	MK819230
	HKAS102261	China	MK804671	MK804651	MK804697	MK819211	MK819231
	HKAS102262	China	MK804672	MK804652	MK804698	MK819212	MK819232
	HKAS102263	China	MK804673	MK804653	MK804699	MK819213	MK819233
	HKAS102264	China	MK804674	MK804654	MK804700	MK819214	MK819234
	HKAS102265	China	MK804675	MK804655	MK804701	MK819215	MK819235
	HKAS102266	China	MK804676	MK804656	MK804702	MK819216	MK819236
	HKAS102267	China	MK804677	MK804657	MK804703	MK819217	MK819237
	NBRC 30312	Japan	AB354982	AB354963			
	NBRC 30147	Japan	AB354981	AB354962			
	NBRC 30737	Japan	AB354983	AB354964			
	GZAAS2.0709	China	GQ845413	KC460983	—	—	—
	GZAAS2.0629	China	GQ845415	KC460980	—	—	—
	GZAAS2.0703	China	GQ845412	KC460981	—	—	—
	GZAAS2.0708 *	China	GQ845414	KC460982	—	—	—
	BJTC HOU999	China, Zhejiang	MZ497363	MZ497378	MZ497389	MZ516171	—
	CNUCC 122	China, Zhejiang	MZ497367	MZ497382	MZ497391	MZ516174	—
	CNUCC 172	China, Zhejiang	MZ497366	MZ497381	MZ497390	MZ516173	—
	CNUCC MJ1	China, Zhejiang	MZ497365	MZ497380	MZ497392	MZ516172	—
*Shiraia* sp.	JP151	Japan	AB255289	AB354977	—	—	—
	JP119	Japan	AB354993	AB354976	—	—	—
	JP185	Japan	AB354994	AB354978	—	—	—
	MSX60519	—	MN970609	—	—	—	—
	slf14	—	GQ355934	—	HM049630	—	—

“—” indicating data unavailable. The strains of new species in this study are emphasized in bold. * Ex-holotype or ex-epitype cultures.

**Table 2:** PCR amplification protocols of this study.

Primer Pairs	Initial Step (T, t)	Denaturation (T, t)	Annealing (T, t)	Elongation (T, t)	Cycles	Final Step (T, t)
ITSIF/ITS4	94 °C, 5 min	94 °C, 30 s	55 °C, 30 s	72 °C, 45 s	35	72 °C, 10 min
LR0R/LR5			55 °C, 30 s	72 °C, 90 s		
NS1/NS4			52 °C, 30 s	72 °C, 90 s		
EF1-983F/EF1-2218R			55 °C, 30 s	72 °C, 60 s		
fRPB2-5f/fRPB2-7cr			50 °C, 30 s	72 °C, 80 s		

**Table 3:** Content of each perylenequinonoid compounds (mg/g) of each fruiting body.

Samples	HA	HB	SA	EA	EB	EC	Total Content
*S. bambusicola* BJTC HOU999	3.60	1.80	4.99	0.00	0.00	0.00	10.39
*R. bambusae* BJTC HOU1000	49.54	6.02	10.34	0.00	0.00	0.00	65.89

**Table 4:** Content of each perylenequinonoid compounds (mg/L) of each culture.

Samples	HA	HB	SA	EA	EB	EC	Total Content
zzz816	84.86	1.71	41.11	226.48	7.19	0.86	362.22
CNUCC 1353PR	677.11	155.36	152.31	326.59	60.41	38.36	1410.13

## Data Availability

Not submitted yet. The sequencing data is prepared to submit to GenBank, the combined alignment and phylogenetic tree are prepared to submit to TreeBASE.

## Supplementary Materials

The following are available online at https://www.mdpi.com/article/10.3390/jof7070563/s1, Table S1: HPLC-DAD-MS data of perylenequinonoid compounds in this study, Figure S1: UV-Vis spectra of HA (A), HB (B), SA (C), EA (D), EB (E), EC (F), the peak in12 min (G), 16 min (H), 17.5 min (I) and 23.6 min (J).
