# Supplementary material for: Phylogenetic Position of Shiraia-Like Endophytes on Bamboos and the Diverse Biosynthesis of Hypocrellin and Hypocrellin Derivatives
Source: J Fungi (Basel). 2021 Jul 14;7(7):563. doi: 10.3390/jof7070563 (PMC8304798; doi:10.3390/jof7070563)
Supplement: Supplementary file 1 [file jof-07-00563-s001.zip › supplementary material/Table S1.pdf]

Table S1. HPLC-DAD-MS data of perylenequinonoid compounds in this study.

Compound	Rt (min)	$[M\text{-}H]^-$ m/z	Molecular Weight
HA	40.3	545.1441	546.52
HB	45.1	527.1368	528.51
SA	36.6	545.1443	546.52
EA	38.3	543.1443	544.51
EB	25.4	545.1450	546.52
EC	21.7	547.1568	548.54
