# Supplementary figures and images for: Phylogenetic Position of Shiraia-Like Endophytes on Bamboos and the Diverse Biosynthesis of Hypocrellin and Hypocrellin Derivatives
Source: J Fungi (Basel). 2021 Jul 14;7(7):563. doi: 10.3390/jof7070563 (PMC8304798; doi:10.3390/jof7070563)

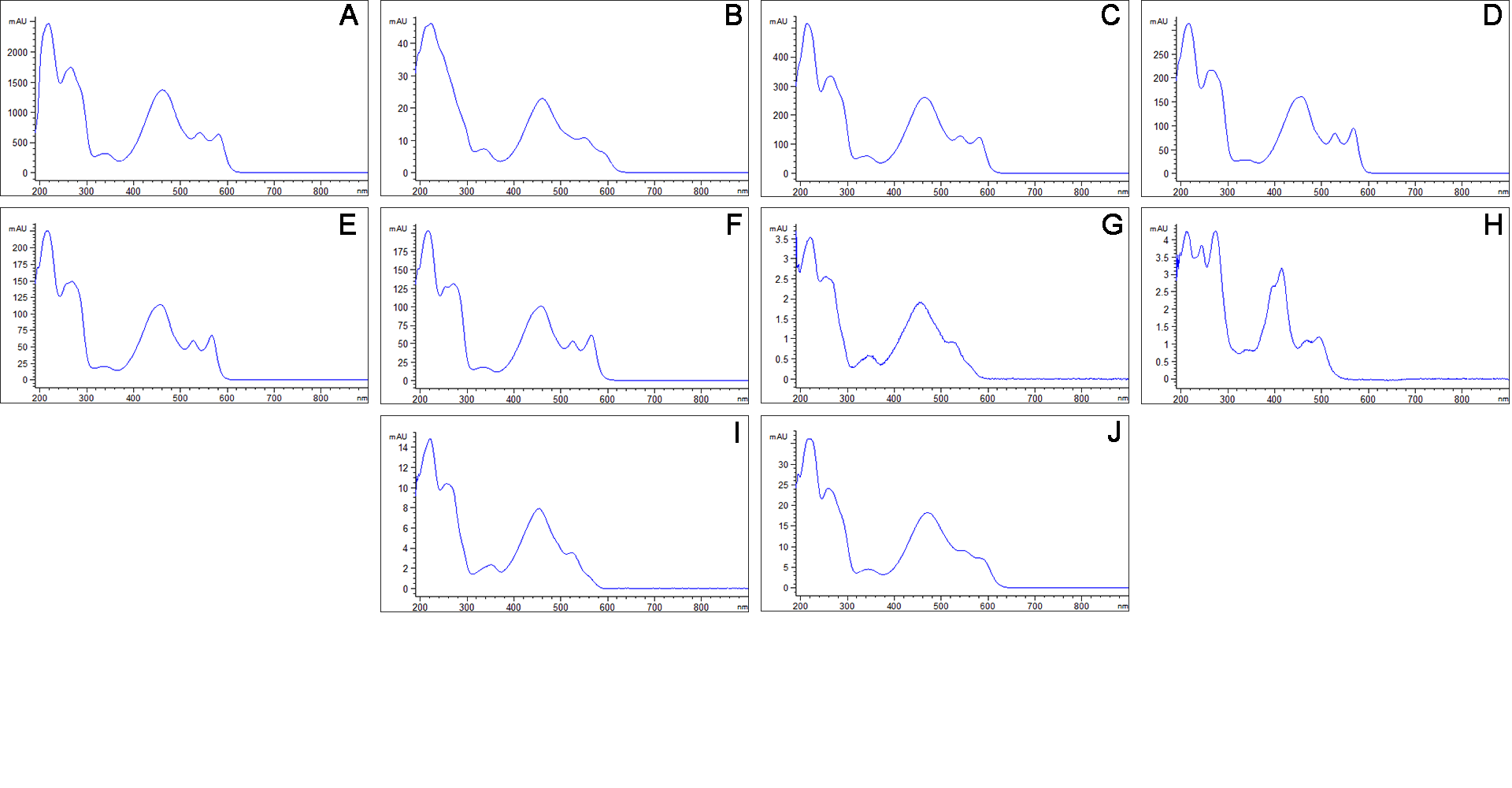

Supplement: Supplementary file 1 [file jof-07-00563-s001.zip › supplementary material/Figure S1.tif]
